# PIM1 in myeloproliferative neoplasms: potential pathogenic and therapeutic implications

**DOI:** 10.1038/s41375-026-03144-5

**Published:** 2026-09-28

**Authors:** Avik Dutta, Lilian Varricchio, Ronald Hoffman, Golam Mohi

**Affiliations:** 1 Department of Biochemistry and Molecular Genetics, University of Virginia School of Medicine, Charlottesville, VA USA; 2 Division of Hematology and Medical Oncology, Tisch Cancer Institute, Icahn School of Medicine at Mount Sinai, New York, NY USA; 3 University of Virginia Comprehensive Cancer Center, Charlottesville, VA USA

**Keywords:** Oncogenesis, Myeloproliferative disease

## Abstract

Myeloproliferative neoplasms (MPNs) are clonal hematologic malignancies characterized by the overproduction of mature myeloid lineage cells. Although JAK2 inhibitors, such as ruxolitinib, can alleviate constitutional symptoms, they typically fail to eradicate malignant clones or reverse bone marrow fibrosis. Moreover, treatment failure and drug intolerance often limit their long-term use. Recent studies have identified PIM1 kinase as an important mediator of MPN pathogenesis. PIM1 expression is upregulated in MPN hematopoietic progenitors, which may contribute to aberrant proliferation and disease progression by regulating key downstream effectors, including mTORC1, BAD, MYC, HIF-1α, and TGF-β. Notably, PIM1 contributes to JAK2 inhibitor-persistent cell growth, and its inhibition restores ruxolitinib sensitivity in JAK2 mutant cells. Preclinical studies using genetic ablation and pharmacological inhibition of PIM1 have shown significant attenuation of the myelofibrosis phenotype in mouse models, providing a strong rationale for targeting this kinase. PIM kinase inhibitors are currently being evaluated in clinical trials for patients with myelofibrosis. This review summarizes the current understanding of PIM1 biology in the context of MPNs, details the molecular mechanisms underlying its pathogenic contributions, and evaluates the translational potential of PIM1-targeted therapies for MPNs.

## Introduction

Myeloproliferative neoplasms (MPNs) are clonal hematopoietic stem cell malignancies characterized by the overproduction of mature myeloid cells across erythroid, megakaryocytic, and granulocytic lineages [1]. The *BCR::ABL1*-negative “classical” MPNs include polycythemia vera (PV), essential thrombocythemia (ET), and myelofibrosis (MF) [1]. While PV and ET are primarily distinguished by erythrocytosis and thrombocytosis, respectively, MF is characterized by progressive bone marrow fibrosis, splenomegaly, ineffective hematopoiesis, dysplastic megakaryocytic hyperplasia, and disabling systemic symptoms [1]. Among these subtypes, MF carries the most unfavorable prognosis, with a median survival of approximately five years and a significant risk of transformation to MPN blast phase (MPN-BP) or secondary acute myeloid leukemia (sAML) [2].

**Fig. 1:**
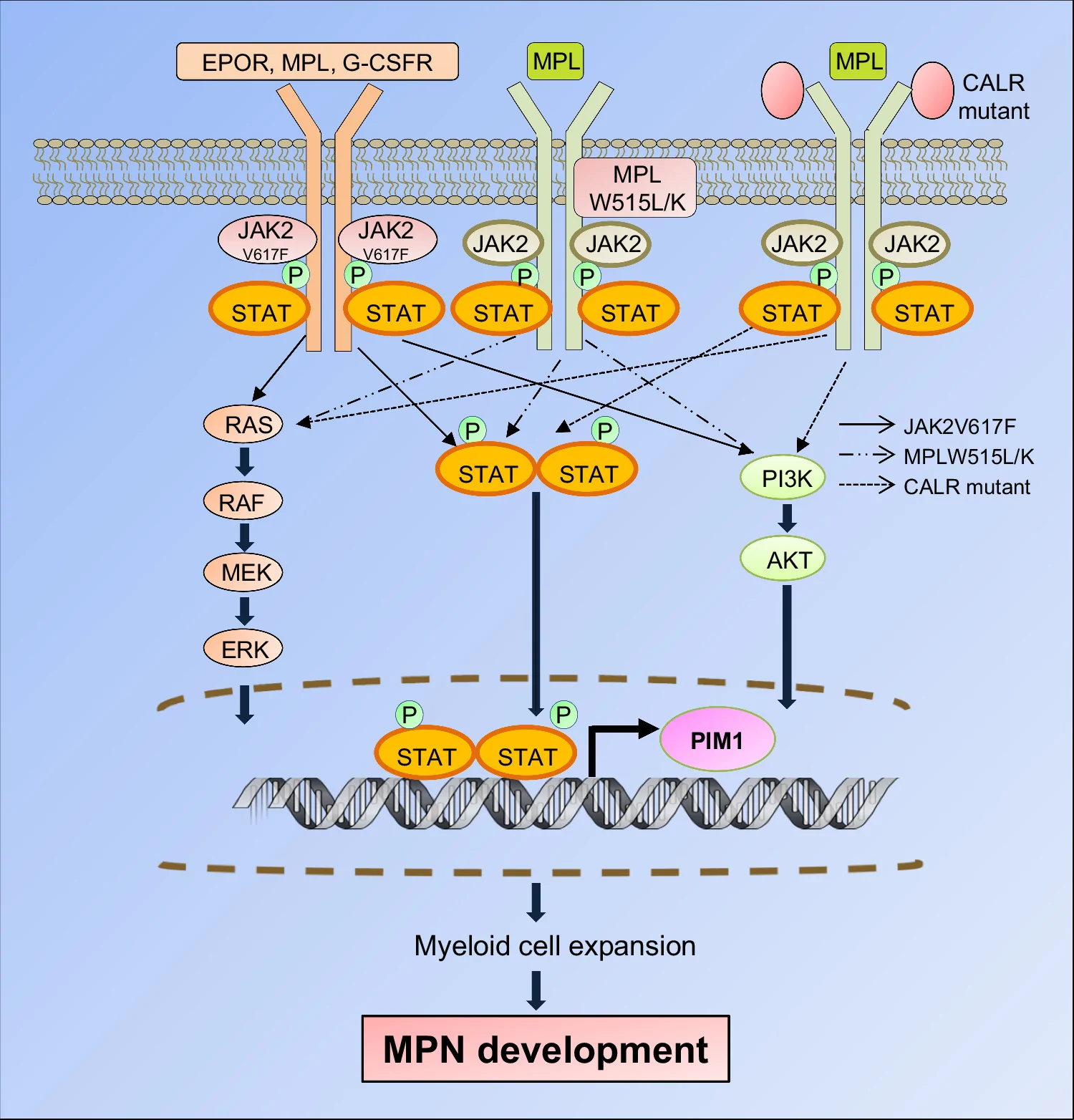
Mechanism of constitutive JAK/STAT signaling activation and PIM1 upregulation in MPN. The JAK2V617F mutant associates with homodimeric type I cytokine receptors (EPOR, MPL, and G-CSFR), prompting ligand-independent receptor dimerization, transphosphorylation, and constitutive activation of STAT, RAS/MAPK, and PI3K/AKT pathways. Similarly, MPL mutations (W515L/K) induce autonomous activation of MPL and its associated JAK2 kinase, driving hyperactivation of JAK/STAT and other downstream signaling cascades. CALR mutants specifically bind to and activate the MPL receptor, triggering constitutive downstream signaling. Constitutive activation of JAK/STAT signaling results in transcriptional upregulation of PIM1, promoting myeloid cell expansion and MPN pathogenesis.

The pathogenesis of MPNs is driven by somatic mutations that induce constitutive downstream signaling. The most prevalent driver is the *JAK2V617F* mutation, detected in ~95% of PV and 50–60% of ET and MF cases [3–6]. This mutation causes constitutive activation of JAK2 tyrosine kinase, which ultimately promotes cytokine-independent hematopoietic cell growth [1, 3, 5]. This process requires the co-expression of type I cytokine receptors, such as the erythropoietin receptor (EPOR), the thrombopoietin receptor (MPL), or the granulocyte colony-stimulating factor receptor (G-CSFR) [7]. Once activated, JAK2V617F induces the activation of the STAT, MEK/ERK, and PI3K/AKT pathways, which collectively contribute to the MPN phenotype (Fig. 1). In vivo expression of the JAK2V617F mutant induces a PV- or ET-like phenotype in mice that progresses to MF in select animal models [8–10].

Somatic mutations in the thrombopoietin receptor gene, *MPL*, are present in 3% to 5% of ET and MF cases [11, 12]. These mutations constitutively activate MPL and its associated JAK2 kinase, inducing activation of downstream signaling pathways [11, 12] (Fig. 1). In vivo expression of the *MPLW515L* mutant causes marked thrombocytosis and the rapid development of MF [11].

Somatic mutations in the calreticulin (*CALR*) are found in 20–30% of ET and MF patients [13, 14]. These mutations typically involve 52-bp deletions or 5-bp insertions in exon 9, causing a frameshift that generates a novel, positively charged C-terminus [13, 14]. The mutant CALR protein binds to and activates MPL in a ligand-independent manner, driving downstream signaling cascades [1] (Fig. 1). Expression of mutant CALR induces an ET-like phenotype in mice that can advance to MF in a subset of animals [15].

Mutations in *JAK2*, *MPL*, and *CALR* are collectively designated as the MPN driver mutations [1]. The causal role of these driver mutations in MPN pathogenesis has been validated through multiple mouse models [8–11, 15]. A unifying molecular hallmark of these three drivers is their constitutive activation of the JAK/STAT signaling [16]. Patients lacking these three drivers, termed “triple-negative MPN”, still exhibit hyperactive JAK/STAT signaling [17], indicating alternative mechanisms of activation. Beyond these primary drivers, co-occurring somatic mutations in epigenetic regulators (*TET2*, *DNMT3A*, *EZH2*, *ASXL1*), splicing factors (*SRSF2*, *U2AF1, SF3B1, ZRSR2*), and *TP53* further alter clonal trajectories and accelerate disease progression [18].

Current therapies for MPNs are largely palliative, except for allogeneic stem cell transplantation, which can be curative; however, its use is restricted by high treatment-related morbidity, advanced patient age, and a lack of optimal stem cell donors [19]. Although the JAK1/2 inhibitor ruxolitinib has transformed the management of MF patients by reducing splenomegaly and constitutional symptoms, it rarely achieves molecular remissions, reverses bone marrow fibrosis, or prolongs survival [20]. Furthermore, the long-term utility of JAK2 inhibitors is constrained by treatment failure and intolerance [20]. Currently, four JAK2 inhibitors are FDA-approved: ruxolitinib, fedratinib, pacritinib, and momelotinib. The choice of a JAK2 inhibitor for a patient with MF is usually determined by their degree of anemia, thrombocytopenia, and treatment tolerance [21]. These limitations highlight the need for additional strategies that target pathways operating in parallel with or downstream of JAK/STAT signaling to more effectively deplete MPN hematopoietic stem/progenitor cells (HSPCs).

Recent studies have identified PIM1, a serine/threonine kinase and direct transcriptional target of JAK/STAT signaling, as a promising therapeutic target in MPNs. PIM1 is overexpressed in MPN HSPCs, collaborates with JAK2 signaling to sustain MPN cell survival, and mediates ruxolitinib persistence/resistance [22–24]. Preclinical studies demonstrate that genetic deletion or pharmacological inhibition of PIM1 ameliorates MPN phenotypes and bone marrow fibrosis in mouse models [22, 25, 26], providing a strong rationale for its therapeutic targeting. This review discusses PIM1 biology, its mechanistic role in MPN pathogenesis, and the current state of PIM inhibitors in clinical development.

## The PIM Kinase Family 

The PIM (Proviral Integration site for Moloney murine leukemia virus) kinase family comprises three closely related serine/threonine kinases: PIM1, PIM2, and PIM3. Unlike most kinases, PIM kinases lack regulatory domains and do not require activation loop phosphorylation for catalytic function [27]. PIM kinases are therefore constitutively active, with their expression regulated primarily at the transcriptional and post-transcriptional levels by cytokines, growth factors, and inflammatory signals [27]. PIM kinases are frequently overexpressed in various hematologic malignancies and solid tumors [27].

*PIM1* was first identified as a proto-oncogene in T-cell lymphoma [28]. The human *PIM1* gene is located on chromosome 6p21.2 and encodes a 313-amino-acid protein. PIM1 kinase exists as two isoforms, PIM1S (34 kDa) and PIM1L (44 kDa), which are generated through alternative translation initiation sites [27]. The human *PIM2* gene is located on chromosome Xp11.23 and encodes a protein sharing 61% amino acid identity with PIM1, whereas *PIM3* maps to 22q13.33 and shares 71% identity with PIM1 at the amino acid level [27, 29]. It has been suggested that PIM kinases may have a compensatory role as well as a context-dependent specific function in cancer [27, 29].

*PIM1* transcription is primarily governed by JAK/STAT signaling [30, 31]. STAT3 and STAT5 transcription factors bind directly to functional response elements within the *PIM1* promoter [31, 32]. Under physiological conditions, PIM1 expression is transiently induced by cytokines and growth factors [33]. However, in malignant cells harboring JAK2, CALR, or MPL mutations, constitutive activation of JAK/STAT signaling drives upregulation of PIM1 expression [16, 22].

PIM1 expression is also regulated by post-transcriptional and post-translational mechanisms. The *PIM1* transcript contains AU-rich elements within its 3’UTR that promote mRNA instability [34], a process modulated by RNA-binding proteins and microRNAs, such as miR-328, miR-486-5p, and miR-124-3p [35–38]. At the protein level, PIM1 turns over rapidly through the ubiquitin-proteasome pathway, with a basal half-life ranging from minutes to hours [39]. The heat shock protein 90 (HSP90) chaperone complex stabilizes PIM1 [39], whereas PP2A-mediated dephosphorylation accelerates its degradation [40]. Oncogenic signaling frequently subverts these degradation pathways, stabilizing PIM1 to sustain its pro-survival effects.

*PIM1*-deficeint mice are viable, fertile, and overtly healthy, displaying only mild erythrocyte microcytosis [41]. In contrast, mice lacking all three PIM kinases (triple-knockout) are viable and fertile but exhibit reduced body size, impaired hematopoietic growth factor responses, decreased platelet counts, and diminished HSPC numbers [29, 42]. This suggests that selective PIM1 inhibition may offer a more favorable therapeutic window than pan-PIM inhibition.

## Downstream targets and functional roles of PIM1

**Fig. 2:**
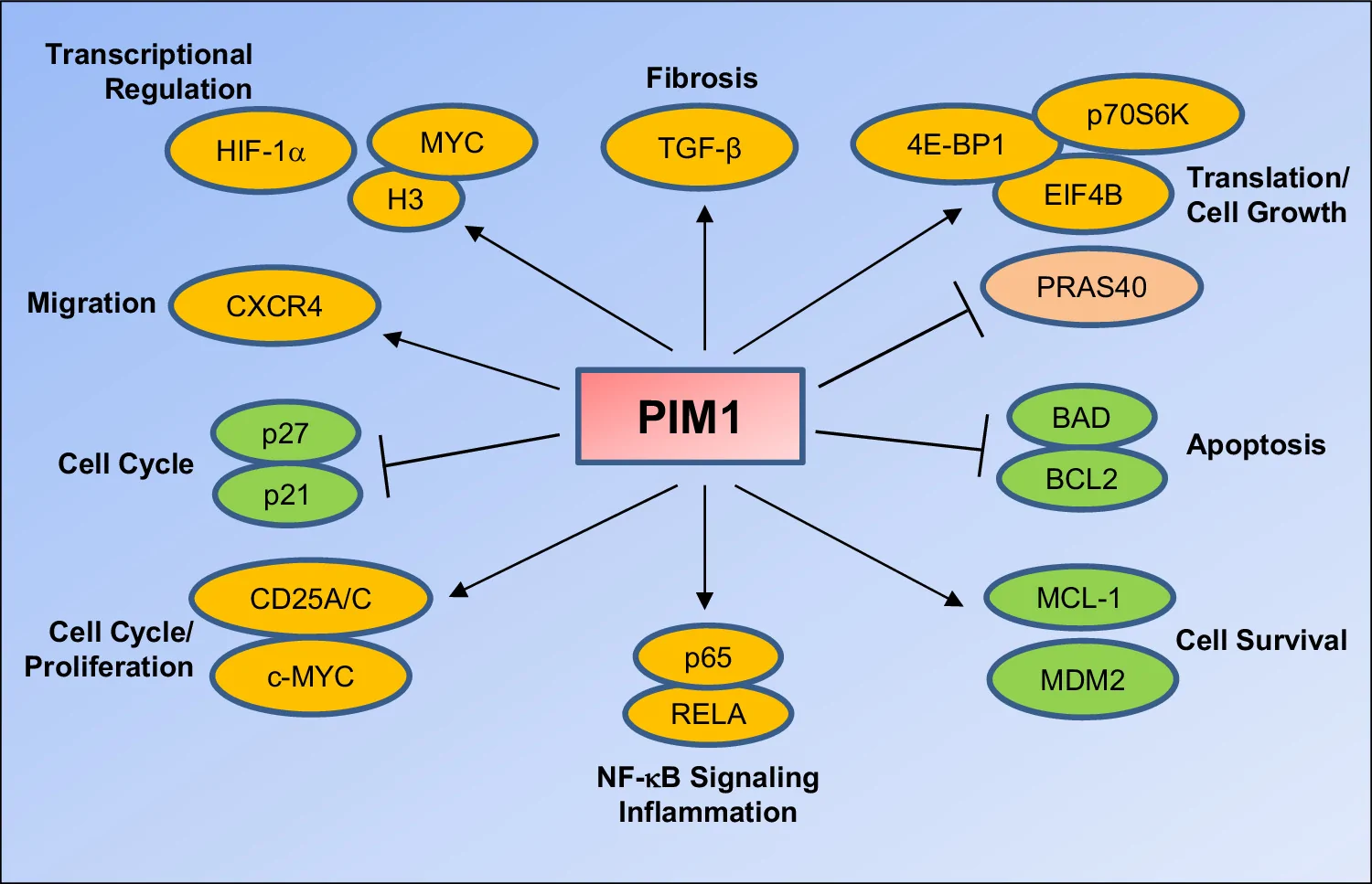
PIM1 targets and biological functions. PIM1 regulates key cellular processes including cell cycle progression, cell survival/apoptosis, protein synthesis, cell migration, inflammation, and tissue fibrosis through phosphorylation and transcriptional regulation of diverse substrates. See text for details.

PIM1 coordinates an oncogenic program by phosphorylating diverse downstream substrates that collectively promote cell survival, cell cycle progression, protein synthesis, metabolic reprogramming, cell migration, inflammation, and tissue fibrosis (Fig. 2).

### Cell survival

PIM1 promotes cell survival through phosphorylation of the pro-apoptotic protein BAD at Ser112 [27, 43]. This prevents BAD from binding and sequestering the anti-apoptotic proteins BCL-2 and BCL-xL, thereby maintaining mitochondrial integrity and averting the apoptotic cascade. Several studies have shown that deletion or inhibition of PIM1 reduces phosphorylation of BAD at Ser112 in JAK2V617F-mutant MPN and AML cells [22, 23, 25, 44]. Furthermore, PIM1 promotes expression of the anti-apoptotic protein MCL-1 in AML and chronic lymphocytic leukemia (CLL) cell lines [45, 46]. Both PIM1 and MDM2 are overexpressed in mantle cell lymphoma, and elevated PIM1 expression correlates with increased MDM2 levels [47]. Furthermore, PIM1 interacts with and phosphorylates MDM2 and induces ARF and p53 expression [47].

### Cell cycle control

PIM1 regulates the cell cycle by phosphorylating key checkpoints at the G1/S and G2/M transitions. It accelerates the G1/S phase transition by phosphorylating the cyclin-dependent kinase (CDK) inhibitors p21 and p27 at multiple residues. Specifically, in U937 leukemia cells, PIM1 phosphorylates p21 (Cip1) at Thr145 to drive its translocation from the nucleus to the cytoplasm [48]. This change in subcellular localization neutralizes the cell cycle inhibitory function of p21. Similarly, PIM1-medited phosphorylation of p27 (Kip1) at Thr157 and Thr198 induces its binding to 14-3-3 protein, resulting in its nuclear export and proteosome-dependent degradation in both solid tumor and leukemia cells [49]. PIM1 is also involved in the transcriptional repression of p27 [49]. Furthermore, PIM1 phosphorylates and activates the phosphatases CDC25A and CDC25C in solid tumor and leukemia cell lines, driving the downstream CDK activation required for cell cycle progression [50, 51].

### MYC oncogene regulation

PIM1 regulates MYC signaling through multiple mechanisms, including transcriptional and post-translational regulation. PIM1-mediated phosphorylation of c-MYC at Ser62 prevents its proteasomal degradation, thereby increasing both protein stability and transcriptional activity, as demonstrated in solid tumor and leukemia cell lines [52]. Stabilized c-MYC sustains a pro-proliferative transcriptional program by upregulating CDKs, cyclins, E2F factors, and CDC25 phosphatases [53, 54]. PIM1 also phosphorylates histone H3 at Ser10 (H3S10) on the nucleosome at MYC-binding sites, contributing to MYC-dependent transcriptional activation and oncogenic transformation in human endothelial and epithelial cell lines [55]. PIM1 is required for the expression of 20% of total MYC target genes [55]. MYC also serves as a master regulator of metabolic reprogramming by directly transactivating glycolytic genes and increasing glucose uptake [56]. Consistent with this, PIM1-mediated phosphorylation of c-MYC activates transcription of key glycolytic genes, including LDHA and PGK1, in ovarian cancer cells [57]. PIM1 silencing diminishes this c-MYC-driven oncogenic program, impairing tumor cell proliferation and survival in prostate and breast cancer cells [58, 59]. PIM1 synergizes with MYC in lymphomagenesis [27]. Furthermore, PIM1 deletion or inhibition reduces the expression of c-MYC in JAK2V617F mutant hematopoietic cells and animal models of MF [22].

### mTORC1 pathway activation

PIM1 activates mTORC1 signaling through direct phosphorylation of PRAS40 at Thr246, an inhibitory component of the mTORC1 complex [60]. This phosphorylation occurs independently of AKT and displaces PRAS40 from the complex, releasing its suppression of mTORC1 catalytic activity [60]. Once active, mTORC1 phosphorylates 4E-BP1 and p70S6 kinase, driving cap-dependent translation and ribosomal biogenesis [61]. PIM1 further promotes translation initiation by phosphorylating eIF4B at Ser406 in leukemia cells [62]. These phosphorylation events on mTORC1 pathway components enhance protein synthesis and support accelerated cell growth.

### Cell migration and homing

PIM1 phosphorylates the chemokine receptor CXCR4 at Ser339, stabilizing CXCR4 on the cell surface and enhancing CXCL12-directed migration and homing of leukemia cells to bone marrow and splenic niches [63, 64]. Pharmacological inhibition of PIM1 reduces surface CXCR4 levels, impairing cell migration and disrupting the microenvironmental crosstalk required for leukemic cell survival in AML and CLL [63, 64]. In advanced MF, however, circulating CD34+ cells show reduced surface CXCR4 despite PIM1 upregulation and JAK2 hyperactivation [65–67].

### Regulation of inflammation

PIM1 and NF-κB act cooperatively to amplify pro-inflammatory signaling. PIM1 phosphorylates the RelA/p65 subunit of NF-κB at Ser276, which prevents its SOCS-1-mediated proteasomal degradation and enhances its transcriptional activity [68]. Conversely, NF-κB acts as a direct transcriptional activator of *PIM1* expression [69]. This coordinated activation loop is further reinforced by the interaction between PIM1 and TAK1, an upstream NF-κB activating kinase [70]. Depletion of PIM1 impairs IKKα/β activation and p65 nuclear translocation in THP-1 cells, suppressing the production of pro-inflammatory cytokines [70].

### Regulation of TGF-β signaling and fibrosis

PIM1 promotes tissue fibrosis by inducing expression of the profibrotic cytokine TGF-β, which triggers extracellular matrix deposition in idiopathic pulmonary fibrosis and MF [22, 71]. Genetic deletion or pharmacological inhibition of PIM1 attenuates SMAD2 phosphorylation and reduces fibrotic progression [22]. PIM1 inhibition also suppresses TGF-β-induced expression of key fibrotic mediators, including collagen, Snail, and αSMA [22, 71]. These findings highlight the PIM1–TGF-β axis as a critical pathway driving tissue fibrosis.

## PIM1 in MPN pathogenesis

Gene expression profiling has shown elevated PIM1 expression in hematopoietic progenitors and granulocytes from MPN patients and JAK2V617F knock-in mice [16, 22, 65]. Although this upregulation occurs across all three classical MPN subtypes, it is most pronounced in MF [16, 22]. Furthermore, PIM1 expression levels positively correlate with the JAK2V617F mutant allele burden, suggesting a dosage-dependent relationship with oncogenic signaling [22]. All three primary MPN driver mutations–*JAK2, CALR*, and *MPL*–induce *PIM1* expression, establishing it as a common downstream effector [22].

Dutta et al. demonstrated that PIM1 plays a non-redundant role in MF. By crossing Pim1 knockout mice with JAK2V617F and MPLW515L models, the authors demonstrated that PIM1 contributes to the development of MF [22]. Deletion of Pim1 significantly reduced leukocytosis, splenomegaly, and bone marrow fibrosis in these models [22]. Notably, Pim2 deletion had no significant effect on MPLW515L-evoked MF, indicating the specific requirement for Pim1 [22]. Pharmacological targeting of PIM1 with TP-3654 (nuvisertib) recapitulated these genetic findings in vivo, normalizing blood counts, reducing splenomegaly, and attenuating bone marrow fibrosis [22]. TP-3654 also preferentially suppressed JAK2V617F-mutant HSPCs over their wild-type counterparts [22]. Notably, TP-3654 alone and in combination with the JAK2 inhibitor ruxolitinib reduced established BM fibrosis, suggesting a disease-modifying effect of PIM1 inhibition in MF [22].

**Fig. 3:**
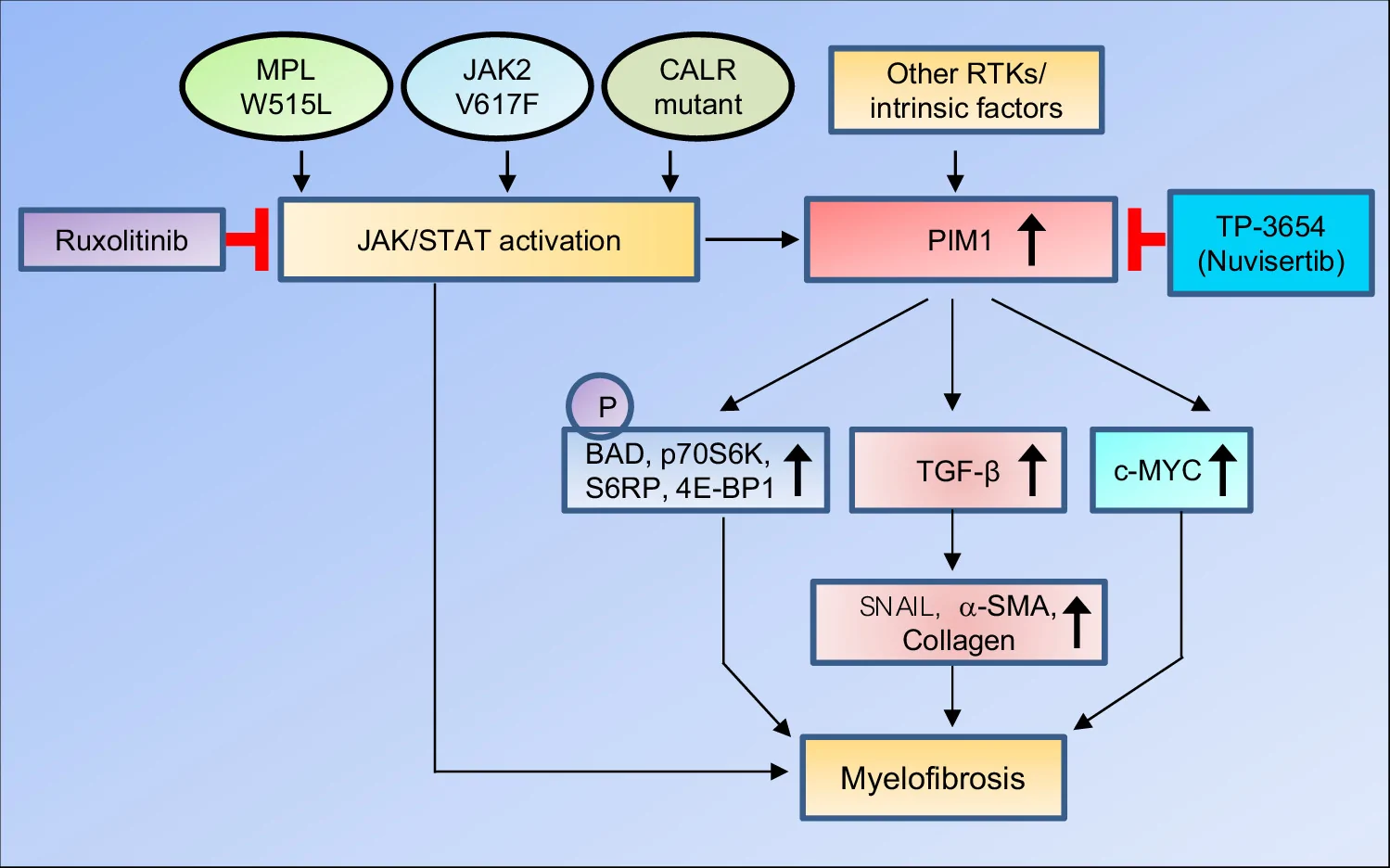
Mechanistic basis for synergy between PIM1 and JAK2 inhibition in myelofibrosis. MPN driver mutations (JAK2, MPL and CALR) trigger constitutive JAK/STAT signaling, which upregulates the transcription of PIM1. PIM1 expression can be further elevated by activation of additional cytokine and grow factor receptor signaling. While ruxolitinib primarily targets STAT signaling, the PIM1 inhibitor TP-3654 (nuvisertib) suppresses the mTORC1, c-MYC, and TGF-β pathways, among others. Furthermore, PIM1 inhibition downregulates key profibrotic effectors, including Collagen, Snail and αSMA. Consequently, dual inhibition of PIM1 and JAK2 produces synergistic suppression of both the proliferative and fibrotic disease components, providing superior therapeutic efficacy in ameliorating myelofibrosis.

Genetic deletion of PIM1 suppresses phosphorylation of S6RP, 4E-BP1, eIF4B, BAD, and SMAD2, and downregulates c-MYC expression in Jak2V617F knock-in mice bone marrow and human HEL cells [22]. Pharmacological inhibition of PIM1 recapitulates these effects, reducing phosphorylation of the same targets and attenuating c-MYC expression in primary MF samples and JAK2V617F mutant hematopoietic cells [22, 24, 25]. Furthermore, PIM1 inhibition reduces TGF-β1 levels and diminishes the expression of key fibrotic effectors, including collagen, Snail, and α-smooth muscle actin (α-SMA) [22]. These findings establish PIM1 as an important mediator of MF pathobiology through its coordinated regulation of mTORC1, c-MYC, and TGF-β signaling, among other pathways (Fig. 3).

Loss of PIM1 has been reported to reduce CXCR4 expression in hematopoietic cells [63]. Consistent with this, elevated PIM1 levels correlate with increased surface CXCR4 expression in both AML and CLL [63, 64]. As previously mentioned, this relationship appears paradoxical in MF, where circulating CD34+ cells exhibit reduced CXCR4 expression despite elevated PIM1 expression [65–67]. This discrepancy is reconciled by the dynamic and stage-dependent regulation of the CXCR4/CXCL12 axis. In early phases of the disease, elevated PIM1 enhances CXCR4 function and promotes clonal expansion within marrow niches. As MF progresses, however, the combination of chronic inflammation, persistent CXCL12 stimulation, constitutive JAK/STAT activation, and elevated proteolytic activity within the fibrotic marrow microenvironment likely induces continuous CXCR4 activation followed by receptor internalization and lysosomal degradation [66, 67, 72]. Consequently, MF cells may retain functionally active CXCR4 signaling downstream of PIM1 while displaying reduced surface CXCR4 expression observed by flow cytometry [66]. We speculate that PIM1 contributes not only to niche retention but also to aberrant trafficking, mobilization, and splenic homing during disease progression. Thus, low detectable surface CXCR4 in advanced MF does not necessarily indicate a loss of CXCR4 pathway dependence; instead, it may reflect chronic receptor engagement and abnormal trafficking associated with constitutive mobilization of malignant CD34+ cells into the peripheral blood and extramedullary sites [66].

## PIM1 signaling and megakaryopoiesis in myelofibrosis

Megakaryocytes (MKs) are central drivers of MF pathobiology [73]. Their increased numbers, dysplastic maturation, and excessive secretion of profibrotic and inflammatory cytokines contribute to bone marrow fibrosis [74]. Despite MK hyperplasia, 20–40% of MF patients develop thrombocytopenia, a complication associated with poor clinical outcomes [75].

Normal megakaryopoiesis requires a precise balance between MK survival and apoptosis. MK progenitors undergo significant cellular stress, including polyploidization and extensive cytoplasmic remodeling, to facilitate platelet production [76, 77]. In MF, hyperactivation of the JAK/STAT pathway upregulates PIM1 and disrupts TPO-mediated MK differentiation [22, 78]. Elevated PIM1 may amplify pro-survival signaling while simultaneously impairing terminal maturation, resulting in ineffective thrombopoiesis. Although anti-apoptotic proteins such as MCL-1 and BCL-xL are essential for MK survival [79], excessive PIM1 activity may sustain an aberrant anti-apoptotic state that favors the persistence of HSPC over functional platelet release. We speculate that PIM1 inhibition restores this equilibrium by eliminating primitive malignant MK progenitors while preserving or reactivating the physiological apoptotic program required for normal platelet production.

PIM1 also supports the inflammatory milieu of MF through modulation of NF-κB [68]. Plasma from thrombocytopenic MF patients exhibits elevated levels of TNF-α and TGF-β1, cytokines known to inhibit MK maturation and worsen thrombocytopenia [80]. LPS stimulation upregulates PIM1 expression along with pro-inflammatory cytokines IL-1β, IL-6 and TNF-α in macrophage-like THP-1 cells, while PIM1 knockdown attenuates LPS-mediated upregulation of these pro-inflammatory cytokines [70]. PIM1 knockdown also reduces LPS-mediated activation of inflammatory signaling pathways, including NF-κB, JNK, and p38 MAPK, in THP-1 cells [70]. Furthermore, PIM1 inhibition reduces TGF-β1 expression in a murine MF model and in classic Hodgkin lymphoma tumor-associated macrophages, and suppresses TNF-α expression in human THP-1 cells [22, 70, 81]. By attenuating these cytokines, PIM1 inhibition may restore MK commitment and terminal differentiation, potentially stabilizing or improving platelet counts in patients.

## PIM1 in MPN disease progression and leukemic transformation

MPNs carry a significant risk of transformation to acute leukemia, designated as MPN blast phase (MPN-BP) or post-MPN sAML. This progression occurs in approximately 20% of patients with MF, 4% with PV and 1% with ET [82]. Beyond sustaining the chronic phase of MPN, PIM1 may also contribute to leukemic transformation. The JAK2V617F–PIM1–HIF-1 regulatory axis, activated through PIM1-mediated stabilization of HIF-1α, provides a direct mechanistic link to leukemic transformation [83]. By reprogramming HIF-1 transcriptional output, this axis generates a gene expression signature (HIF1-MPN-BP) that correlates with disease severity, progression rate, and transformation risk [83]. Notably, PIM1 expression level correlates with disease severity in MPN, and its inhibition represses HIF1-MPN-BP gene expression changes in JAK2V617F-mutant leukemia cells [83]. Early intervention with PIM1 inhibitors may therefore offer a dual benefit: controlling chronic-phase symptoms while delaying or preventing leukemic progression.

In post-MPN sAML, PIM1 expression remains high and is regulated by multiple oncogenic pathways. Bromodomain and extra-terminal domain (BET) inhibition reduces PIM1 protein levels in sAML cells [84], while CDK7 targeting depletes PIM1 alongside MYC and CDK4/6 [85]. This depletion reduces sAML cell viability and exhausts the leukemic stem/progenitor compartment in vivo. Co-targeting CDK7 and BET induces synergistic lethality, indicating that PIM1 remains a therapeutic vulnerability from early-stage MPN through advanced blast-phase disease.

## PIM1 in other hematologic malignancies

PIM1 expression is elevated not only in MPNs but across a broad spectrum of hematologic malignancies, suggesting its important role in malignant hematopoiesis. Early studies demonstrated PIM1 overexpression in approximately 30% of human myeloid and lymphoid leukemias [86].

PIM1 is frequently upregulated in AML, particularly in FLT3-ITD-positive cases [87]. FLT3-ITD-mediated activation of STAT5 induces PIM1 overexpression, which confers resistance to PI3K/Akt inhibitors by enhancing the mTORC1/Mcl-1 pathway in AML cell lines [88]. PIM1 is also a direct transcriptional target of *HOXA9*, a gene commonly dysregulated in AML [89]. High PIM1 levels independently predict lower remission rates, shorter survival, and greater risk of relapse in AML [90].

In diffuse large B-cell Lymphoma (DLBCL), PIM1 is overexpressed in most cases and correlates with STAT3/STAT5 activation and disease progression [91]. *PIM1*, along with *MYC*, *ARHH*, and *PAX5*, is also a recurrent target of aberrant somatic hypermutation in more than 50% of DLBCL cases [92]. *PIM1* mutations are more common in the ABC subtype of DLBCL, where they enhance clonogenic growth and promote resistance to ibrutinib [93].

In CLL and mantle cell lymphoma (MCL), PIM1 carries both prognostic and functional significance [64, 94]. Elevated PIM1 expression independently predicts poor outcomes for MCL patients undergoing intensive therapy [94]. In multiple myeloma (MM), PIM1 and PIM2 are overexpressed and contribute to proliferation, survival, cell cycle dysregulation, and bone destruction [95]. IL-6/STAT3 and NF-κB signaling sustain constitutive PIM2 expression, reducing myeloma cell dependence on exogenous growth factors [95].

In T-cell acute lymphoblastic leukemia (T-ALL), somatic mutations in the JAK/STAT pathway drive aberrant PIM1 expression [96, 97]. PIM inhibition reduces mutant cell growth and suppresses c-MYC and mTOR pathways in mutant cells, indicating PIM1 as a therapeutic vulnerability in T-ALL [97]. PIM1 is constitutively active in adult T-cell leukemia/lymphoma (ATL), acting as a primary effector of STAT3, which is mutated in 25% of primary ATL samples [98]. ATL cells are particularly sensitive to PIM inhibitors, supporting the STAT3–PIM1 axis as a therapeutic target in this disease [98].

## PIM1 and JAK2 inhibitor persistence in MPN

Multiple lines of evidence indicate that PIM1 is a driver of ruxolitinib persistence/resistance in MPNs. While PIM1 expression is upregulated in JAK2V617F mutant hematopoietic cells, its expression is further elevated in ruxolitinib-persistent JAK2V617F mutant cells [22]. Exogenous overexpression of PIM1 in JAK2V617F-mutant cells is sufficient to confer ruxolitinib persistence/resistance [22, 23]. Conversely, pharmacological PIM inhibition restores ruxolitinib sensitivity in persistent MPN cell models [22, 23]. This re-sensitization is mechanistically linked to the restoration of BAD-mediated pro-apoptotic signaling and the suppression of mTOR signaling, pathways typically sustained by aberrant PIM1 activity [22, 23, 25].

*PIM1* transcription is driven primarily by JAK/STAT signaling but is also regulated by NF-κB and c-MYC [27, 55, 69]. PIM1 protein may persist and continue signaling even after a JAK2 inhibitor such as ruxolitinib has suppressed JAK2 phosphorylation, providing a plausible molecular route by which MPN clones tolerate JAK2 inhibition. Koppikar et al., showed that MPN cells re-establish JAK-STAT signaling through JAK1/JAK2 or TYK2/JAK2 heterodimers despite continued JAK2 inhibitor exposure, allowing malignant cells to persist rather than being eradicated [99]. Dual inhibition of JAK2 and PIM1 therefore represents a promising strategy to overcome JAK2 inhibitor persistence and achieve more durable clinical responses.

## Therapeutic targeting of PIM1 in MPN

The development of PIM kinase inhibitors has progressed through several generations. Early pan-PIM inhibitors, including SGI-1776 and AZD1208, showed promising pre-clinical activity against AML and MPN cells [23, 24, 44, 45]. SGI-1776 is an imidazopyridazine compound with nanomolar activity against all three PIM kinases (IC_50_ values of 7 nM, 363 nM, and 69 nM for PIM1, PIM2, and PIM3, respectively) [46]. SGI-1776 also potently inhibits FLT3 (IC_50_, 44 nM) [46]. AZD1208 is a potent inhibitor of all three PIM kinases, with IC_50_ values of 0.4 nM for PIM1, 5 nM for PIM2, and 1.9 nM for PIM3 [44]. These agents suppressed hematopoietic colony formation and induced apoptosis by activating BAD and suppressing the mTOR pathway [23, 44]. However, early clinical development of these inhibitors was hindered by poor safety and pharmacokinetic profiles. SGI-1776 was discontinued due to dose-limiting cardiac toxicity (QTc prolongation), while AZD1208 failed to produce significant responses in Phase I AML trials [100] (Table 1). AZD1208’s failure resulted from dose-limiting gastrointestinal toxicity and drug-induced CYP3A4 activity, which increased drug clearance and prevented sustained therapeutic concentrations [100].

**Table 1:** List of PIM kinase inhibitors used in clinical trials for MPNs and myeloid leukemia.

Drug	Disease	Clinical trial identification	Clinical stages and observations
SGI-1776	Relapsed/ refractory leukemia	NCT01239108	Phase I; discontinued due to cardiac QTc prolongation
AZD1208	AML	NCT01489722	Phase I; terminated due to lack of clinical efficacy and increased CYP3A4 activity
PIM447	MF	NCT02370706	Phase I; discontinued due to severe hematologic toxicities
INCB053914	MF, AML, MDS	NCT02587598	Phase I; terminated due to poor clinical response
TP-3654 (Nuvisertib)	Relapsed/ refractory MF	NCT04176198	Phase I/II ongoing; favorable safety profile, promising clinical activity

*MPN* myeloproliferative neoplasms, *MF* myelofibrosis, *AML* acute myeloid leukemia, *MDS* myelodysplastic neoplasia, *QTc* corrected QT interval, *CYP3A4* cytochrome P450 3A4.

INCB053914 is a pan-PIM kinase inhibitor with IC_50_ values of 0.24 nM for PIM1, 30 nM for PIM2, and 0.12 nM for PIM3 [101]. INCB053914 showed efficacy in multiple preclinical MPN models, synergizing with ruxolitinib to inhibit MPN cell growth, induce apoptosis, and suppress colony-formation of primary MPN HSPCs [25, 101]. In vivo, this compound reduced ruxolitinib-persistent myeloproliferation [25]. However, in a Phase I/II trial (NCT02587598), INCB053914 showed no efficacy as monotherapy and provided limited clinical benefit when combined with ruxolitinib in patients with MF; hepatotoxicity (ALT and AST elevations) was the primary adverse event [102].

PIM447 (LGH447) is a pan-PIM inhibitor with greater inhibitory potency than first-generation compounds, with K_i_ values of 6 pM, 18 pM, and 9 pM for PIM1, PIM2, and PIM3, respectively [103]. It also inhibits GSK3β, PKN1, and PKCτ [103]. A triple-combination approach using PIM447, ruxolitinib, and the CDK4/6 inhibitor ribociclib induced apoptosis in JAK2V617F-mutant cells, reduced splenomegaly and bone marrow fibrosis, and prolonged survival in murine MPN models [26]. These findings are consistent with Dutta et al., who showed that combined CDK4/6 and JAK2 inhibition significantly reduced splenomegaly and bone marrow fibrosis in MF models [104]. However, PIM447 has faced challenges in clinical translation. A Phase I trial of PIM447 in MM was discontinued due to low single-agent activity and significant hematologic toxicity [105]. A Phase I study of the triple combination of PIM447, ruxolitinib, and ribociclib in MF (NCT02370706) was completed in 2020, but further evaluation has not been reported, likely due to severe dose-limiting hematologic toxicity encountered during the trial.

TP-3654 (nuvisertib) is a second-generation PIM kinase inhibitor with greater selectivity for PIM1 over PIM2 and PIM3 (IC_50_ values of 5 nM for PIM1, 239 nM for PIM2, and 42 nM for PIM3) [106]. It shows at least 10-fold selectivity for PIM1 over any other kinases tested [106]. It also shows superior preclinical activity compared with first-generation PIM inhibitors [106]. In murine JAK2V617F and MPLW515L MF models, TP-3654 monotherapy normalized blood counts, reduced splenomegaly, and attenuated bone marrow fibrosis [22]. Combined with ruxolitinib, TP-3654 produced greater reductions across all disease parameters compared to either agent alone, with near-complete abrogation of bone marrow fibrosis [22]. TP-3654 also preferentially targeted JAK2V617F-mutant over wild-type HSPCs, reflecting therapeutic selectivity for the malignant clone. These findings supported the initiation of an ongoing multicenter Phase I/II clinical trial (NCT04176198) in patients with MF, including those refractory to or intolerant of ruxolitinib (Table 1). Preliminary data presented at the 2024 and 2025 ASH Annual Meetings showed that TP-3654 was well-tolerated, with limited myelosuppression [107, 108].

Initial efficacy signals for TP-3654 are promising, including symptomatic relief, modulation of circulating cytokines, improvements in anemia and thrombocytopenia, and reductions in bone marrow fibrosis grade [107, 108]. The lower toxicity and greater in vivo efficacy of TP-3654 (nuvisertib) compared with other Pan-PIM inhibitors may reflect its greater selectivity for PIM1. Based on these outcomes, the trial has been expanded to include combination cohorts pairing TP-3654 (nuvisertib) with ruxolitinib or momelotinib.

Since anemia and thrombocytopenia represent critical unmet needs in MF patients and are frequently exacerbated by ruxolitinib, combining TP-3654 (nuvisertib) with momelotinib offers a distinct therapeutic rationale, as momelotinib has been reported to improve anemia in MF patients [109]. Some studies indicate that momelotinib inhibits the ACVR1 receptor, suppressing hepcidin expression and improving hemoglobin levels, while another study suggests that its inhibition of ACVR1 is only modest [109–111]. The combination of TP-3654 (nuvisertib) with momelotinib has been evaluated in a phase I/II clinical trial (NCT04176198). All 18 patients enrolled in this study had intermediate- or high-risk MF and had relapsed after or were refractory to JAK2 inhibitor therapy. At baseline, participants had hemoglobin levels <10 g/dL, platelet counts ≥50 × 10^9^/L, measurable splenomegaly, and constitutional symptoms. Median treatment duration was 21 weeks. The combination therapy was well tolerated, with only one patient experiencing a dose-limiting toxicity (Grade 4 thrombocytopenia without bleeding). Treatment-related adverse events occurring in ≥20% of patients included diarrhea, nausea, and thrombocytopenia. Mean hemoglobin levels and platelet counts remained stable throughout the 24-week treatment period. At week 24, a ≥25% spleen volume reduction was observed in 40% of patients, and systemic symptom improvement occurred in 100% of patients (60% achieved a ≥50% reduction in total symptom score). In addition, 40% of patients showed anemia improvement over the 24 weeks of treatment: one patient showed a hemoglobin response (defined as a mean increase of ≥1.0 g/dL for ≥12 weeks without transfusion), and another achieved a >50% reduction in transfusion burden. These preliminary data support further development of the nuvisertib/momelotinib combination in MF [112]. Recognizing the urgent need for new MF treatments, the FDA granted Fast Track Designation to TP-3654 (nuvisertib) in June 2025 for patients with intermediate- or high-risk MF.

## Conclusions and future directions

PIM1 kinase plays a pivotal role in MPN pathobiology. Downstream of oncogenic JAK/STAT signaling, its constitutive overexpression shifts PIM1 from a transient physiological regulator to a persistent driver of malignant cell survival, clonal expansion, and disease progression. PIM1 may contribute to the diverse clinical manifestations of MPNs by modulating an effector network that stabilizes MYC, activates mTORC1, suppresses BAD-dependent apoptosis, reprograms HIF-1α, and promotes profibrotic TGF-β signaling.

The role of PIM1 in sustaining ruxolitinib-persistent cells provides a strong rationale for its clinical evaluation. Preclinical models have validated PIM1 as an important mediator of MPN pathogenesis [22], while early clinical data indicate that combined PIM1 and JAK2 inhibition is both feasible and well-tolerated [112]. By targeting persister/resistant cells, PIM1 inhibition could yield deeper molecular responses than JAK2 inhibitor monotherapy alone. Future studies should focus on three areas: (1) identifying predictive biomarkers of PIM1 inhibitor sensitivity in patients; (2) optimizing combinations of PIM1 inhibitors with JAK2, BET, or mTOR inhibitors through preclinical and translational studies; and (3) evaluating PIM1 inhibition in high-risk populations, specifically patients with ruxolitinib-refractory MF and post-MPN sAML.
